# Segregation of the mouse germline and soma

**DOI:** 10.1080/15384101.2019.1672466

**Published:** 2019-10-04

**Authors:** Man Zhang, Ian Chambers

**Affiliations:** MRC Centre for Regenerative Medicine, Institute for Stem Cell Research, School of Biological Sciences, University of Edinburgh, Edinburgh, Scotland

**Keywords:** Primordial germ cell, germline competent, formative pluripotency, transcription factors, OTX2, NANOG

## Abstract

Mouse primordial germ cells (PGCs), originate from the early post-implantation epiblast in response to BMP4 secreted by the extraembryonic ectoderm. However, how BMP4 acts here has remained unclear. Recent work has identified the transcription factor (TF), OTX2 as a key determinant of the segregation of the germline from the soma. OTX2 is expressed ubiquitously in the early post-implantation epiblast, decreasing rapidly in cells that initiate the PGC programme. *Otx2* mRNA is also rapidly repressed by BMP4 *in vitro*, in germline competent cells. Supporting a model in which BMP4 represses *Otx2*, enforcing sustained OTX2 expression in competent cells blocks germline entry. In contrast, *Otx2*-null epiblast cells enter the germline with increased efficiency *in vitro* and *in vivo* and can do so independently of BMP4. Also, *Otx2*-null cells can initiate germline entry even without the crucial PGC TF, BLIMP1. In this review, we survey recent advances and propose hypotheses concerning germline entry.

## Introduction

Two decades ago, Lawson et al showed that BMP4 originating in the extraembryonic ectoderm was required to induce primordial germ cell (PGC) formation in the adjacent epiblast of the early post-implantation mouse embryo [1]. Subsequent analyses have shown that upon removal of the overlying endoderm, most, if not all, epiblast cells can undergo germline specification in response to BMP4 [2]. Although further studies have identified many of the transcription factors (TFs) that influence PGC specification [3–6], how BMP4 signaling into the proximal epiblast caused germline specification has remained unclear. Here we review recent work identifying the TF OTX2 as a key determinant of the choice between a germline and somatic fate and assess some of the implications of this finding.

Some of the earliest single cell cDNA studies in mammalian cells were focused on early germline development and analyzed cDNA libraries prepared from single epiblast cells [3,7,8]. This identified Fragilis, an interferon-inducible transmembrane protein, as a marker of the onset of germ cell development. Shortly thereafter, *Blimp1* was identified as one of the earliest TFs of the germline [4]. BLIMP1 appears to be responsible for both the repression of somatic gene expression and for the full activation of the germline programme [9]. Subsequently, *Prdm14* was shown mainly to activate some germline specific markers [5], while, *Ap2γ* both activated germline-specific markers and repressed somatic mRNAs[6]. These three TFs form a tripartite gene regulatory network (GRN) that determines germline fate in mice [10,11].

Our ability to study early germline development took a giant leap forward when Hayashi et al. demonstrated how to reconstitute mouse PGC specification *in vitro*[12], thereby circumventing problems due to the limited access to the mouse peri-implantation embryo. In this protocol, embryonic stem cells (ESCs), previously cultured in the presence of inhibitors of MEK and GSKb (2i)[13] were switched into medium containing βFGF and ActivinA for two days. Under these conditions ESCs progress to a more advanced pluripotent state, referred to as epiblast-like cells (EpiLCs). Transcriptionally, these EpiLCs resemble epiblast cells of the peri-implantation embryo. Moreover, EpiLCs are competent to enter the germline and can give rise to PGC-like cells (PGCLCs) which express the primordial germ cell TFs, *Blimp1, Prdm14* and *Ap2γ*. To make the transition to PGCLCs, it is absolutely essential that wild type EpiLCs are switched into culture media containing BMP4 [12]. Interestingly, the cell surface antigens, SSEA-1 and CD61 were found to mark these emergent PGCLCs leading to their use as surrogate markers for PGCLC identity[12]. Remarkably, these *in vitro*-derived PGCLCs could yield functional sperm and eggs after transplantation into the gonads [12,14]. This PGCLC differentiation system opened the door to investigations of the mechanisms underlying entry of pluripotent cells into the germline. This showed that the combined induction of *Blimp1, Prdm14* and *Ap2*γ in EpiLCs could circumvent the need for exposure to BMP4 for acquisition of a PGCLC identity [11]. Furthermore, subsequent work showed that induction of the TF NANOG, best known for its role in stimulating efficient ESC self-renewal [15,16], could also enable BMP4-independent acquisition of a PGCLC identity. However, these studies did not tell us how BMP4 signaling results in expression of the PGC TF genes *Blimp1, Prdm14* and *Ap2*γ. Recent results have shown that the TF OTX2 is a key intermediary acting between BMP4 signaling and PGC TF gene induction at the point when cells choose to either develop a somatic identity or enter the germline [17].

## Otx2 downregulation precedes the germ cell initiation both in vitro and in vivo

In the past 12 years, it has become apparent that pluripotent cells do not just exist as naïve ESCs in culture. Rather pluripotent cells can adopt a range of states, developing from a naïve ESC state through a so-called formative EpiLC state to a primed epiblast stem cell (EpiSC) state [18,19]. These states are considered to most closely reflect a developmental transition *in vivo* from a pre-implantation epiblast identity [20] to an early post-implantation epiblast (E5.75) identity [12] to a post-implantation anterior epiblast identity[21].

OTX2 is a homeodomain protein with a well characterized role as an anterior neurectoderm determinant [22,23]. More recently *Otx2* has been shown to be expressed in pluripotent cells. Specifically, *Otx2* is expressed in formative pluripotent cells and is involved in the transition between naïve and formative pluripotent states by redirecting OCT4 to new binding sites in chromatin [24–26]. Intriguingly, OTX2 and the naïve pluripotency TF NANOG possess antagonistic functions in ESCs cultured in LIF/FCS, in which a spectrum of pluripotent states can co-exist [27]. Until recently however, the dynamic expression of *Otx2* during PGC initiation from pluripotent cells was unknown. Analysis of mRNA expression at the beginning of PGCLC differentiation *in vitro* showed that *Otx2* mRNA was widely expressed in formative cells and was downregulated rapidly after EpiLCs were moved into PGCLC media [17]. Notably, this is 12-24h before the mRNAs encoding the key PGC TFs, *Blimp1, Prdm14* and *AP2*γ begin to increase. Furthermore, at the single cell level, BLIMP1 and AP2γ proteins only become detectable in cells in which OTX2 protein is strongly downregulated[17].

Importantly, this spatio-temporal relationship between changes in expression of OTX2 and PGC TFs also holds *in vivo*. During mouse development, at E5.5, the epiblast has lost NANOG expression [28] and expresses OTX2 ubiquitously[17]. At E6.5, OTX2 becomes downregulated specifically in a subset of epiblast cells that show incipient expression of early germline markers, FRAGILIS and BLIMP1 [17]. By E7.5, the epiblast contains a coherent domain of cells in which OTX2 is undetectable that coincides with the domain of cells in which BLIMP1 is clearly expressed [17]. Together these results suggested the hypothesis that OTX2 may negatively regulate entry of cells into the germline.

## Otx2 deletion enhances germline entry efficiency both in vivo and in vitro

Consistent with a repressive function of OTX2 on germline entry, *Otx2^−/-^* cells generate approximately 10 times more PGCLCs than *Otx2^+/+^* cells *in vitro* [17]. With respect to the underlying mechanism, *Otx2^−/-^* cells activate expression of PGC TF genes quicker and to a greater extent than *Otx2^+/+^* cells. In particular, *Ap2γ* and *Nanog* mRNAs become expressed within six hours of changing *Otx2*^−/-^ cells to PGCLC differentiation medium, some three hours earlier than *Blimp1* and *Prdm14* mRNAs. This suggests that AP2γ could lie upstream of *Blimp1*, at least in the absence of OTX2. Interestingly, OTX2 binds directly to potential *cis* regulatory elements near *Blimp1, Prdm14* and *Ap2γ* [25]. However, a direct assessment of the effect of OTX2 binding on the function of these putative regulatory elements remains to be performed.

A repressive function of OTX2 on PGC specification efficiency is also supported by *in vivo* analysis. While *Otx2^−/-^* ESCs formed chimaeric mice with a comparable efficiency to *Otx2^+/+^* cells, the germline contribution efficiency was higher in *Otx2^−/-^* than in *Otx2^+/+^* chimaeras with some chimaeras having all the *Otx2^−/-^* cells confined to the germline [17]. In addition, although *Otx2^−/-^* embryos showed strong development defects, these embryos had a twofold increase in PGC numbers at E7.5 compared to the wild type heterozygous littermates [17].

## Otx2 overexpression blocks entry to the germline

**Figure 1.**
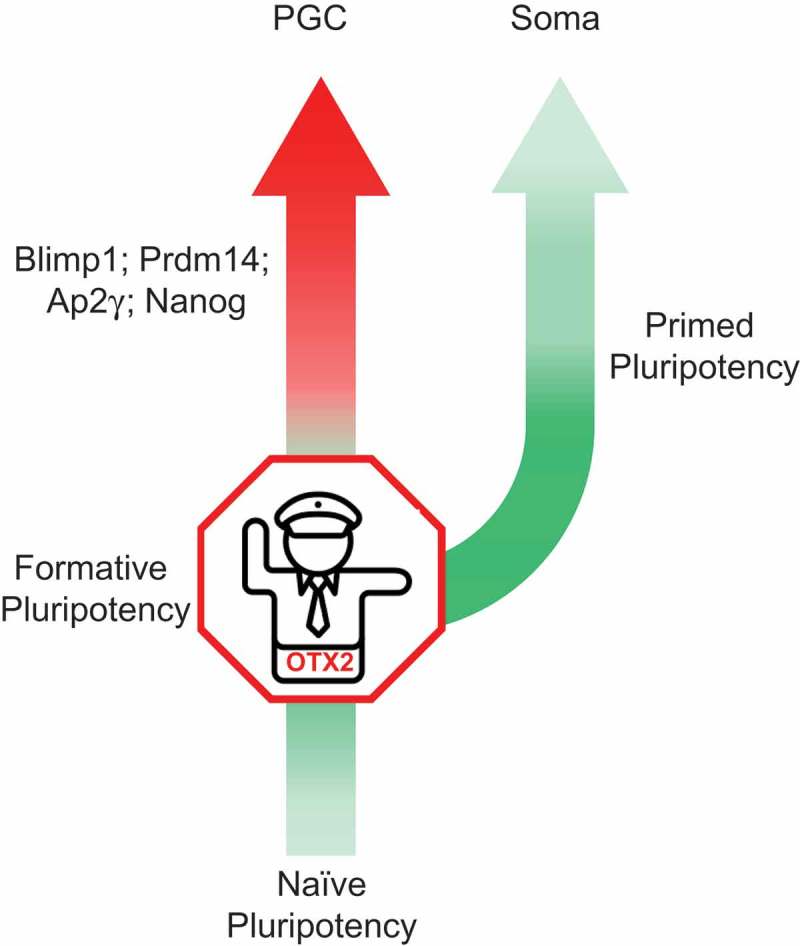
OTX2 acts like a traffic warden to restrict the entry of cells to the germline and usher them instead toward primed pluripotency and ultimately to a somatic fate.

*In vivo*, epiblast cells remain competent to enter germline differentiation for a limited time [2]. *In vitro*, OTX2 overexpression for the first two days of PGCLC induction blocked germ cell differentiation completely. Together with results from *Otx2^−/-^* cells mentioned above, this suggests a model in which OTX2 acts like a traffic warden to restrict the entry of cells to the germline and usher them instead toward a somatic fate (Figure 1). However, this traffic warden-like function is time-limited since after day 2, when cells have become specified to a germ cell fate, OTX2 overexpression had no effect on PGCLC differentiation. This indicates that OTX2 acts to inhibit the initial stage of PGC differentiation but does not interfere with the function of an established germline gene regulatory network. These results also support a model of germline entry in which BMP4 induces PGCs by suppressing *Otx2* expression. Temporal mRNA analysis confirmed this hypothesis, by showing that BMP4 accelerated the rate of decrease of *Otx2* mRNA expression, such that after 24 hours of BMP4 treatment *Otx2* mRNA was 20% of the starting level, whereas without BMP4 it remained at 60%. This analysis also highlights a role for endogenous wnt signaling during PGC development [29]. Addition of a wnt antagonist to the PGCLC differentiation media had no effect on the early suppression of *Otx2* mRNA but blocked the continued decrease after 12 hours. This suggests that endogenous wnt signaling acts downstream of BMP4 in *Otx2* suppression in agreement with the activation of *wnt3* in isolated epiblasts cultured in the presence of BMP4 [30]. It will be interesting to see whether exogenous wnt can enhance the effects of BMP4 on *Otx2* suppression and germline entry.

## Otx2 deletion enables cells to enter the germline without requiring cytokines or BLIMP1

Cytokines are essential for PGCLC induction in wild type cells [12]. If the critical role of BMP4 in PGCLC induction is the suppression of *Otx2* mRNA, then *Otx2^−/-^* EpiLCs should differentiate toward PGCLCs without requiring BMP4. Indeed, this is the case [17]. Moreover, while *Blimp1* is crucial for the germline entry of wild type cells [4], *Otx2^−/-^* cells could initiate germline entry and activate some aspects of germline fate in cells in which the PGC “master regulator” *Blimp1* had been deleted. The principal function of BLIMP1 in PGC induction is to repress somatic gene expression [9,31]. However, *Otx2*-null cells did not activate expression of the somatic genes *T, Hoxa1* or *Hoxb1* when placed in PGCLC inducing media, indicating that an inability to activate the somatic programme may be one way in which *Otx2*^−/-^ cells circumvent a requirement for BLIMP1. Despite an ability to express CD61 and SSEA-1, *Otx2*^−/-^; *Blimp1*^−/-^ do not develop a transcriptional profile appropriate to PGCLCs, indicating a need for BLIMP1 function for full PGCLC identity in an *Otx2*-null background.

## Questions for the future

**Figure 2.**
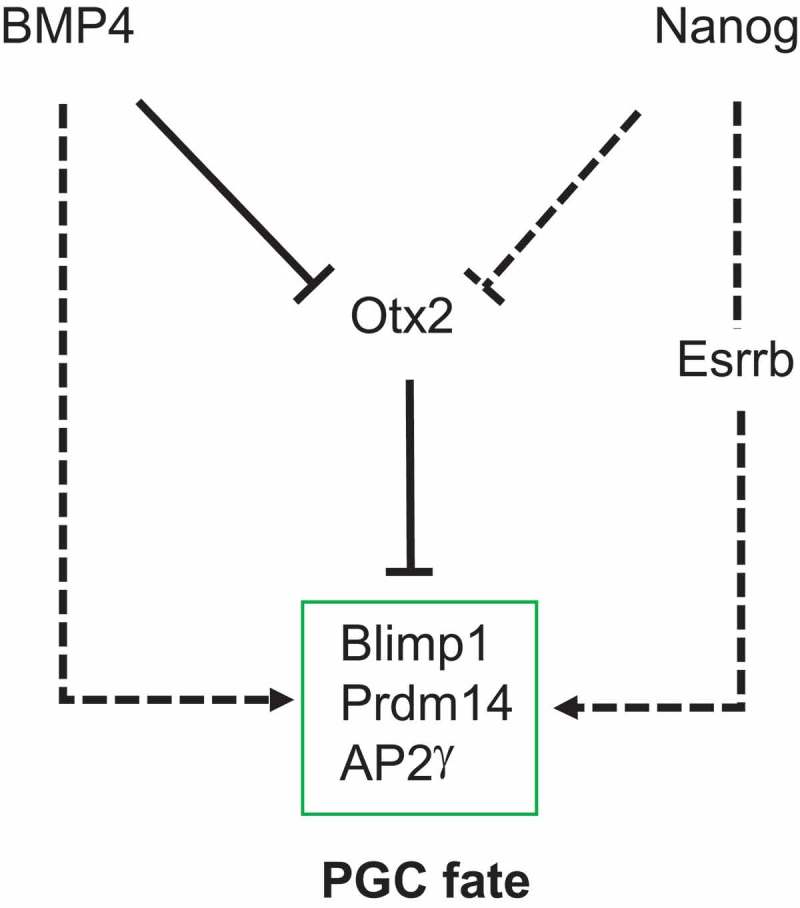
The underlying molecular events for PGC specification. Activating interactions are indicated by arrows and repressive interactions by blunt arrows. Putative relationships that remain to be fully established during the early events in germline specification are indicated by broken lines.

While the above studies establish a central role for *Otx2* in communication between BMP4 signaling and expression of the PGC TFs, they also raise several questions. For example, *Otx2^−/-^* cells can undergo PGCLC differentiation in the absence of BMP4. However, if the only function of BMP4 was to suppress Otx2 expression, then the efficiency of PGCLC differentiation of *Otx2*^−/-^ cells should be identical either in the presence or absence of cytokines. However, while >70% of *Otx2*^−/-^ cells become CD61^+^/SSEA1^+^ during PGCLC differentiation, this drops to 30% in the absence of cytokines. This implies that, in addition to *Otx2* repression, PGCLC cytokines may operate an OTX2-independent pathway(s) to further stimulate germline entry (Figure 2). Identification of this putative OTX2-independent function of BMP4 is a key focus of future studies. Modulation of this pathway may enable *Otx2*^+/+^ cells to enter the germline independently of cytokines. Alternatively, the action of this hypothetical pathway may only be detectable in *Otx2*^−/-^ cells. In this case we may hypothesize that combining modulation of this hypothetical pathway with *Otx2* deletion could enable 100% of cells to enter the germline in the absence of cytokines.

The data referred to above showed that *Otx2*^−/-^:*Blimp1*^−/-^ cells can enter the germline (as assessed by CD61/SSEA1 and activation of an Oct4 reporter transgene) but their PGCLC transcriptome goes awry prior to day 6 of PGCLC differentiation. Although it is uncertain what the primary defect is in *Otx2*^−/-^:*Blimp1*^−/-^ cells, future RNA-seq time course analysis should clarify this. It will also be interesting to determine whether defects in *Otx2*^−/-^ PGCLCs become apparent when *Ap2*γ or *Prdm14* are deleted. If such defects do occur then understanding their nature will provide a better picture of the interdependencies between TFs of the germline gene regulatory network, which will in turn enhance our understanding of the transcriptional control of PGC identity.

**Figure 3.**
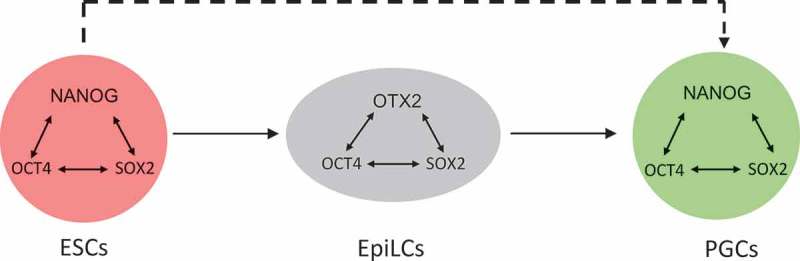
Pluripotency gene regulatory network changes during PGC differentiation. The central TFs of the pluripotency gene regulatory network in distinct cell states are indicated by double-headed arrows. During transition from ESCs to EpiLCs NANOG is extinguished and OTX2 is expressed. Upon transition from EpiLCs to PGCLCs OTX2 is extinguished and NANOG is re-expressed. The broken line connecting ESCs to PGCLCs indicates the hypothetical possibility that ESCs may transit to PGCLCs without going through an EpiLC state.

Pluripotency exists as a developmental continuum between the naïve pre-implantation state and the primed post-implantation state from which effective lineage determination occurs. Between the two states is considered to be the formative pluripotency state for which EpiLCs are the *in vitro* representative [19]. However, while some naïve pluripotency TFs are absent, little is known about what functions of the pluripotency GRN are required in the formative state. Work from Buecker et al. showed that OTX2 becomes expressed during the naïve to EpiLC transition and that OTX2 redirects OCT4 binding to chromatin during the transition from the naïve to the formative state [25]. However, mutant cells lacking OTX2 enter the germline from an EpiLC state with high efficiency, even in the absence of the essential cytokine signals. This indicates that the EpiLC state differs in the presence or absence of OTX2, in agreement with reported differences in gene expression[25]. EpiLCs are both competent for germline entry and capable of forming differentiated somatic cells of the three primary germ layers. However, while these properties may theoretically be held by the same cells at the same time, it is important to recognize that these properties are not identical. Therefore, it may be possible to enter the germline without passing through the formative state (Figure 3). This underscores the importance of fully characterizing the essential, defining attributes of the optimal germline competent state.

**Figure 4.**
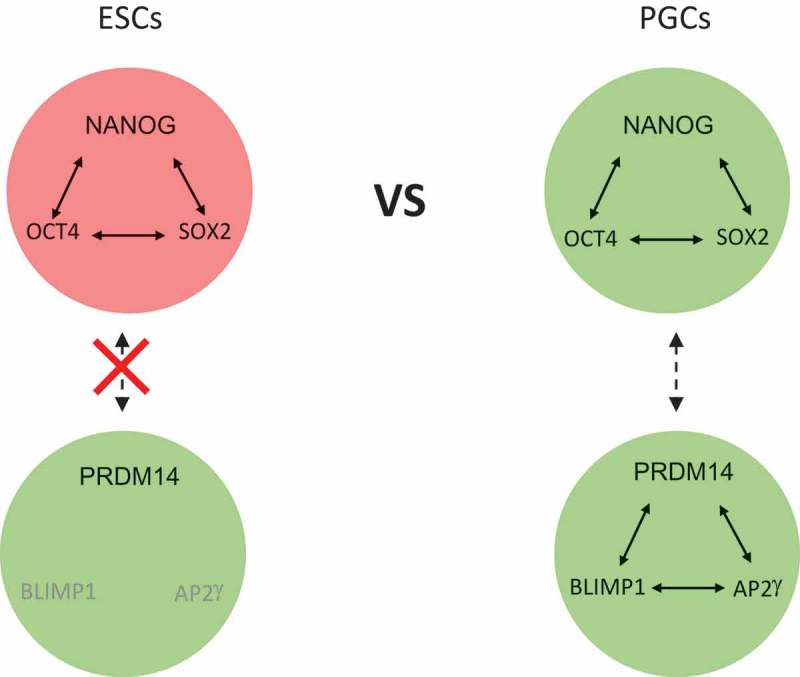
Distinctions between gene regulatory networks in ESCs and PGCs. Both cell types express OCT4, SOX2 and NANOG. While ESCs also express PRDM14, they do not express the PGC TFs BLIMP1 and AP2γ. The precise nature of the connection between the OCT4, SOX2, NANOG network and the PGC network in PGCs remains to be fully established. A key unresolved question is why ESCs do not express all the PGC network components.

Pluripotency TFs have roles during PGC development [32–34]. In addition, regulatory interactions by pluripotency TFs on genes encoding the PGC TFs have been reported [35]. Although *Prdm14* is a downstream target of NANOG that is expressed in ESCs[36], *Blimp1* and *Ap2*γ are not expressed in ESCs (Figure 4). Why Oct4, Sox2, Nanog and Prdm14 are expressed in both ESCs and PGCs but *Blimp1* and *Ap2*γ are absent from ESCs is an outstanding unresolved question. In addition, the precise roles played by pluripotency TFs during segregation of the germline remain to be established. For example, enhancers directing *Oct4* expression differ in naïve and primed pluripotent cells [37], and differences in putative enhancer occupancy by OCT4 in formative cells have been reported [25]. However, a detailed understanding of how OCT4 influences enhancer function during the transition through a germline competent state remains to be established. Another question concerns the role of NANOG. In common with some other pluripotency TFs expressed in naïve pre-implantation cells, NANOG is not expressed during peri-implantation development [15,38], nor in EpiLCs [12,28]. Previous work has demonstrated that NANOG and OTX2 work antagonistically in ESCs [27,39] and OTX2 has been shown to be robustly expressed in both EpiLCs and the peri-implantation epiblast [17,24]. However, OTX2 is not responsible for ongoing repression of *Nanog* in EpiLCs [17]. Notably, when NANOG expression is enforced in EpiLCs, PGCLC differentiation no longer requires cytokines [34,35]. This similarity to the *Otx2*^−/-^ phenotype suggests that the NANOG overexpression phenotype may be due to a direct suppression of *Otx2*. However, although studies in the embryo indicate that immediately after implantation, all epiblast cells express OTX2 and that subsequently OTX2 is specifically extinguished in cells that go on to express BLIMP1, the timing of re-expression of NANOG with respect to OTX2 and BLIMP1 is currently unclear. Murakami et al have proposed that NANOG acts by binding to the regulatory elements of *Blimp1* and *Prdm14* in competent cells to activate their expression. This would be consistent with a model in which re-expression of NANOG represses *Otx2* and activates *Blimp1* and *Prdm14*. However, binding of NANOG to *Blimp1* and *Prdm14* regulatory elements was measured in EpiLCs following two days of overexpression of NANOG. Further work is therefore required to understand where the primary functional role for endogenous NANOG in PGCLC induction occurs.

It has also been reported that *Esrrb* is a direct target gene of NANOG in ESCs [34,36]. While ESRRB can substitute for NANOG function during PGC development *in vivo* [34,36], our unpublished data suggests that ESRRB cannot fully substitute for NANOG function in germline induction *in vitro*. Therefore, the extent to which ESRRB is necessary for PGCLC differentiation induced by NANOG needs to be further addressed (Figure 2). Notably, in ESCs, NANOG negatively regulates *Otx2* but ESRRB does not [36]. It will therefore be interesting to determine whether reducing the *Otx2* gene dosage is sufficient to enable ESRRB induction to drive EpiLCs into PGCLC differentiation in the absence of BMP4.

## Conclusions

In the last two decades, knowledge from mouse and non-human primate embryos combined with the recently established *in vitro* PGCLC differentiation system has shown that cytokine signaling can induce critical germline-specific TFs and initiate germline entry. However, deletion of the TF OTX2 is sufficient to enable cells to enter to germline in the absence of cytokine signals. This suggests that cells can obtain a germline fate simply by avoiding a somatic fate, an idea with profound evolutionary implications [17,40,41]. This could occur by removing OTX2, a putative component of the formative GRN and a negative regulator of germline entry. Such a strategy of removing a negative regulator of a particular cell fate option (in this case the germline) could be a generally applicable mechanism to drive cell fate decisions, as opposed to induction of a positive regulator. Finally, current evidence suggests that early germline development is regulated distinctly in human and mouse [42–46]. This raises the question of whether OTX2 works as a traffic warden to restrict germline entry in humans, as it does in the mouse (Figure 1). If not, it will be of interest to determine whether there is another factor that acts similarly to mouse OTX2 to restrict PGC development in humans.

## References

[1] Lawson KA, Dunn NR, Roelen BA, et al Bmp4 is required for the generation of primordial germ cells in the mouse embryo. Genes Dev. 1999;13:424–436. 10049358 10.1101/gad.13.4.424 PMC316469

[2] Ohinata Y, Ohta H, Shigeta M, et al A signaling principle for the specification of the germ cell lineage in mice. Cell. 2009;137:571–584. 19410550 10.1016/j.cell.2009.03.014

[3] Saitou M, Barton SC, Surani MA. A molecular programme for the specification of germ cell fate in mice. Nature. 2002;418:293–300. 12124616 10.1038/nature00927

[4] Ohinata Y, Payer B, O’Carroll D, et al Blimp1 is a critical determinant of the germ cell lineage in mice. Nature. 2005;436:207–213. 15937476 10.1038/nature03813

[5] Yamaji M, Seki Y, Kurimoto K, et al Critical function of Prdm14 for the establishment of the germ cell lineage in mice. Nat Genet. 2008;40:1016–1022. 18622394 10.1038/ng.186

[6] Weber S, Eckert D, Nettersheim D, et al Critical function of AP-2 gamma/TCFAP2C in mouse embryonic germ cell maintenance. Biol Reprod. 2010;82:214–223. 19776388 10.1095/biolreprod.109.078717

[7] Yabuta Y, Kurimoto K, Ohinata Y, et al Gene expression dynamics during germline specification in mice identified by quantitative single-cell gene expression profiling. Biol Reprod. 2006;75:705–716. 16870942 10.1095/biolreprod.106.053686

[8] Tanaka SS, Nagamatsu G, Tokitake Y, et al Regulation of expression of mouse interferon-induced transmembrane protein like gene-3, Ifitm3 (mil-1, fragilis), in germ cells. Dev Dyn. 2004;230:651–659. 15254899 10.1002/dvdy.20085

[9] Kurimoto K, Yabuta Y, Ohinata Y, et al Complex genome-wide transcription dynamics orchestrated by Blimp1 for the specification of the germ cell lineage in mice. Genes Dev. 2008;22:1617–1635. 18559478 10.1101/gad.1649908 PMC2428060

[10] Magnusdottir E, Dietmann S, Murakami K, et al A tripartite transcription factor network regulates primordial germ cell specification in mice. Nat Cell Biol. 2013;15:905–915. 23851488 10.1038/ncb2798 PMC3796875

[11] Nakaki F, Hayashi K, Ohta H, et al Induction of mouse germ-cell fate by transcription factors in vitro. Nature. 2013;501:222–226. 23913270 10.1038/nature12417

[12] Hayashi K, Ohta H, Kurimoto K, et al Reconstitution of the mouse germ cell specification pathway in culture by pluripotent stem cells. Cell. 2011;146:519–532. 21820164 10.1016/j.cell.2011.06.052

[13] Ying QL, Wray J, Nichols J, et al The ground state of embryonic stem cell self-renewal. Nature. 2008;453:519–523. 18497825 10.1038/nature06968 PMC5328678

[14] Hayashi K, Ogushi S, Kurimoto K, et al Offspring from oocytes derived from in vitro primordial germ cell-like cells in mice. Science. 2012;338:971–975. 23042295 10.1126/science.1226889

[15] Chambers I, Colby D, Robertson M, et al Functional expression cloning of Nanog, a pluripotency sustaining factor in embryonic stem cells. Cell. 2003;113:643–655. 12787505 10.1016/s0092-8674(03)00392-1

[16] Chambers I, Silva J, Colby D, et al Nanog safeguards pluripotency and mediates germline development. Nature. 2007;450:1230–1234. 18097409 10.1038/nature06403

[17] Zhang J, Zhang M, Acampora D, et al OTX2 restricts entry to the mouse germline. Nature. 2018;562:595–599. 30283136 10.1038/s41586-018-0581-5 PMC6485399

[18] Nichols J, Smith A Naive and primed pluripotent states. Cell Stem Cell. 2009;4:487–492. 19497275 10.1016/j.stem.2009.05.015

[19] Smith A Formative pluripotency: the executive phase in a developmental continuum. Development. 2017;144:365–373. 28143843 10.1242/dev.142679 PMC5430734

[20] Boroviak T, Loos R, Bertone P, et al The ability of inner-cell-mass cells to self-renew as embryonic stem cells is acquired following epiblast specification. Nat Cell Biol. 2014;16:516–528. 24859004 10.1038/ncb2965 PMC4878656

[21] Kojima Y, Kaufman-Francis K, Studdert JB, et al The transcriptional and functional properties of mouse epiblast stem cells resemble the anterior primitive streak. Cell Stem Cell. 2014;14:107–120. 24139757 10.1016/j.stem.2013.09.014

[22] Ang SL, Jin O, Rhinn M, et al A targeted mouse Otx2 mutation leads to severe defects in gastrulation and formation of axial mesoderm and to deletion of rostral brain. Development. 1996;122:243–252. 8565836 10.1242/dev.122.1.243

[23] Acampora D, Mazan S, Lallemand Y, et al Forebrain and midbrain regions are deleted in Otx2-/- mutants due to a defective anterior neuroectoderm specification during gastrulation. Development. 1995;121:3279–3290. 7588062 10.1242/dev.121.10.3279

[24] Acampora D, Di Giovannantonio LG, Simeone A Otx2 is an intrinsic determinant of the embryonic stem cell state and is required for transition to a stable epiblast stem cell condition. Development. 2013;140:43–55. 23154415 10.1242/dev.085290

[25] Buecker C, Srinivasan R, Wu Z, et al Reorganization of enhancer patterns in transition from naive to primed pluripotency. Cell Stem Cell. 2014;14:838–853. 24905168 10.1016/j.stem.2014.04.003 PMC4491504

[26] Yang SH, Kalkan T, Morissroe C, et al Otx2 and Oct4 drive early enhancer activation during embryonic stem cell transition from naive pluripotency. Cell Rep. 2014;7:1968–1981. 24931607 10.1016/j.celrep.2014.05.037 PMC4074343

[27] Acampora D, Di Giovannantonio LG, Garofalo A, et al Functional antagonism between OTX2 and NANOG specifies a spectrum of heterogeneous identities in embryonic stem cells. Stem Cell Reports. 2017;9:1642–1659. 29056334 10.1016/j.stemcr.2017.09.019 PMC5935799

[28] Hoffman JA, Wu CI, Merrill BJ Tcf7l1 prepares epiblast cells in the gastrulating mouse embryo for lineage specification. Development. 2013;140:1665–1675. 23487311 10.1242/dev.087387 PMC3621485

[29] Aramaki S, Hayashi K, Kurimoto K, et al A mesodermal factor, T, specifies mouse germ cell fate by directly activating germline determinants. Dev Cell. 2013;27:516–529. 24331926 10.1016/j.devcel.2013.11.001

[30] Ben-Haim N, Lu C, Guzman-Ayala M, et al The nodal precursor acting via activin receptors induces mesoderm by maintaining a source of its convertases and BMP4. Dev Cell. 2006;11:313–323. 16950123 10.1016/j.devcel.2006.07.005

[31] Kurimoto K, Yabuta Y, Hayashi K, et al Quantitative dynamics of chromatin remodeling during germ cell specification from mouse embryonic stem cells. Cell Stem Cell. 2015;16:517–532. 25800778 10.1016/j.stem.2015.03.002

[32] Kehler J, Tolkunova E, Koschorz B, et al Oct4 is required for primordial germ cell survival. EMBO Rep. 2004;5:1078–1083. 15486564 10.1038/sj.embor.7400279 PMC1299174

[33] Campolo F, Gori M, Favaro R, et al Essential role of Sox2 for the establishment and maintenance of the germ cell line. Stem Cells. 2013;31:1408–1421. 23553930 10.1002/stem.1392

[34] Zhang M, Leitch HG, Tang WWC, et al Esrrb complementation rescues development of Nanog-null germ cells. Cell Rep. 2018;22:332–339. 29320730 10.1016/j.celrep.2017.12.060 PMC5775501

[35] Murakami K, Gunesdogan U, Zylicz JJ, et al NANOG alone induces germ cells in primed epiblast in vitro by activation of enhancers. Nature. 2016;529:403–407. 26751055 10.1038/nature16480 PMC4724940

[36] Festuccia N, Osorno R, Halbritter F, et al Esrrb is a direct Nanog target gene that can substitute for Nanog function in pluripotent cells. Cell Stem Cell. 2012;11:477–490. 23040477 10.1016/j.stem.2012.08.002 PMC3473361

[37] Yeom YI, Fuhrmann G, Ovitt CE, et al Germline regulatory element of Oct- 4specific for the totipotent cycle of embryonal cells. Development. 1996;122:881–894. 8631266 10.1242/dev.122.3.881

[38] Osorno R, Tsakiridis A, Wong F, et al The developmental dismantling of pluripotency is reversed by ectopic Oct4 expression. Development. 2012;139:2288–2298. 22669820 10.1242/dev.078071 PMC3367440

[39] Heurtier V, Owens N, Gonzalez I, et al The molecular logic of Nanog-induced self-renewal in mouse embryonic stem cells. Nat Commun. 2019;10:1109. 30846691 10.1038/s41467-019-09041-z PMC6406003

[40] Johnson AD, Alberio R Primordial germ cells: the first cell lineage or the last cells standing?. Development. 2015;142:2730–2739. 26286941 10.1242/dev.113993 PMC4550962

[41] Extavour CG, Akam M Mechanisms of germ cell specification across the metazoans: epigenesis and preformation. Development. 2003;130:5869–5884. 14597570 10.1242/dev.00804

[42] Kobayashi T, Zhang H, Tang WWC, et al Principles of early human development and germ cell program from conserved model systems. Nature. 2017;546:416–420. 28607482 10.1038/nature22812 PMC5473469

[43] Irie N, Weinberger L, Tang WW, et al SOX17 is a critical specifier of human primordial germ cell fate. Cell. 2015;160:253–268. 25543152 10.1016/j.cell.2014.12.013 PMC4310934

[44] Tang WW, Dietmann S, Irie N, et al A unique gene regulatory network resets the human germline epigenome for development. Cell. 2015;161:1453–1467. 26046444 10.1016/j.cell.2015.04.053 PMC4459712

[45] Sasaki K, Yokobayashi S, Nakamura T, et al Robust in vitro induction of human germ cell fate from pluripotent stem cells. Cell Stem Cell. 2015;17:178–194. 26189426 10.1016/j.stem.2015.06.014

[46] Chen D, Liu W, Zimmerman J, et al The TFAP2C-regulated OCT4 Naive enhancer is involved in human germline formation. Cell Rep. 2018;25:3591–602 e5. 30590035 10.1016/j.celrep.2018.12.011 PMC6342560

